# Thin PDMS Films Using Long Spin Times or Tert-Butyl Alcohol as a Solvent

**DOI:** 10.1371/journal.pone.0004572

**Published:** 2009-02-24

**Authors:** John H. Koschwanez, Robert H. Carlson, Deirdre R. Meldrum

**Affiliations:** Department of Electrical Engineering, University of Washington, Seattle, Washington, United States of America; Center for Genomic Regulation, Spain

## Abstract

Thin polydimethylsiloxane (PDMS) films are frequently used in “lab on a chip” devices as flexible membranes. The common solvent used to dilute the PDMS for thin films is hexane, but hexane can swell the underlying PDMS substrate. A better solvent would be one that dissolves uncured PDMS but doesn't swell the underlying substrate. Here, we present protocols and spin curves for two alternatives to hexane dilution: longer spin times and dilution in tert-butyl alcohol. The thickness of the PDMS membranes under different spin speeds, spin times, and PDMS concentrations was measured using an optical profilometer. The use of tert-butyl alcohol to spin thin PDMS films does not swell the underlying PDMS substrate, and we have used these films to construct multilayer PDMS devices.

## Introduction

Polydimethylsiloxane (PDMS), an elastomer widely used in microdevice fabrication [1], is frequently spun into membranes for use as a flexible component of valves [2], [3], actuators [4], and microlenses [5]. The membrane is normally integrated into the device in one of two ways: (i) PDMS is spin-coated onto a glass or silicon wafer and then lifted off the wafer by peeling it up with another piece of PDMS, or (ii) PDMS is spin-coated directly onto the final PDMS, glass, or silicon substrate.

**Figure 1:**
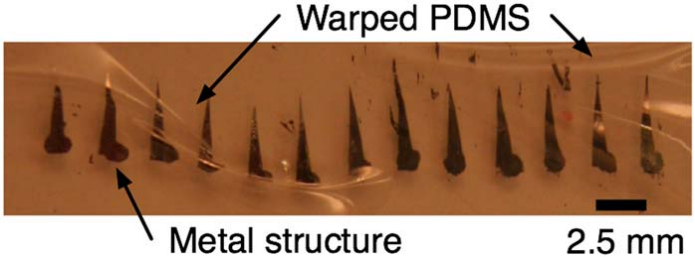
PDMS membrane that was warped when hexane-diluted PDMS was spun onto it. The triangular elements are metal that was plated on the underlying PDMS membrane.

Uncured PDMS is often diluted in solvent in order to spin thin (<5 µm) films. Hexane is often used as a solvent for uncured PDMS [4], [6], but hexane swells cured PDMS. If the substrate layer is a PDMS membrane, the membrane can warp. In our case, we required a thin layer of PDMS to cover microfabricated ferromagnetic elements in a multilayer PDMS device [7]. When we used hexane to dilute the PDMS, the magnets were warped and unusable (Figure 1). The ideal solvent for uncured PDMS is one that dilutes the uncured PDMS, but does not swell cured PDMS membranes.

Here we propose two alternatives to hexane dilution. The first alternative, longer spin times (>5 min), is an obvious solution, but PDMS film thickness data for long spin times is not widely available. The second alternative is the use of tert-butyl alcohol (TBA) as a solvent for uncured PDMS. TBA dilutes uncured PDMS, but does not swell cured PDMS membranes. We present thickness data for various concentrations of PDMS in TBA for various spin speeds.

## Results

We tested 12 different solvents for their ability to: (1) dissolve uncured PDMS, and (2) not visibly warp a cured PDMS membrane when poured directly onto the membrane. Tert-butyl alcohol (TBA) was the only solvent that met both requirements. We also measured the swelling in the thickness of cured PDMS soaked in water, TBA, and hexane to dry PDMS (Table 1). The slight amount of swelling (1.05) in TBA was acceptable in our device construction.

**Table 1:** Swelling in the thickness of cured PDMS after soaking in 3 different solvents for 2 hours.

Solvent	Ratio of thickness after soak to thickness before soak
Water (23°C)	1.00
Tert-butyl alcohol (45°C)	1.05
Hexane (23°C)	1.31

Standard deviation divided by mean in all 3 cases was less than 1%.

**Figure 2:**
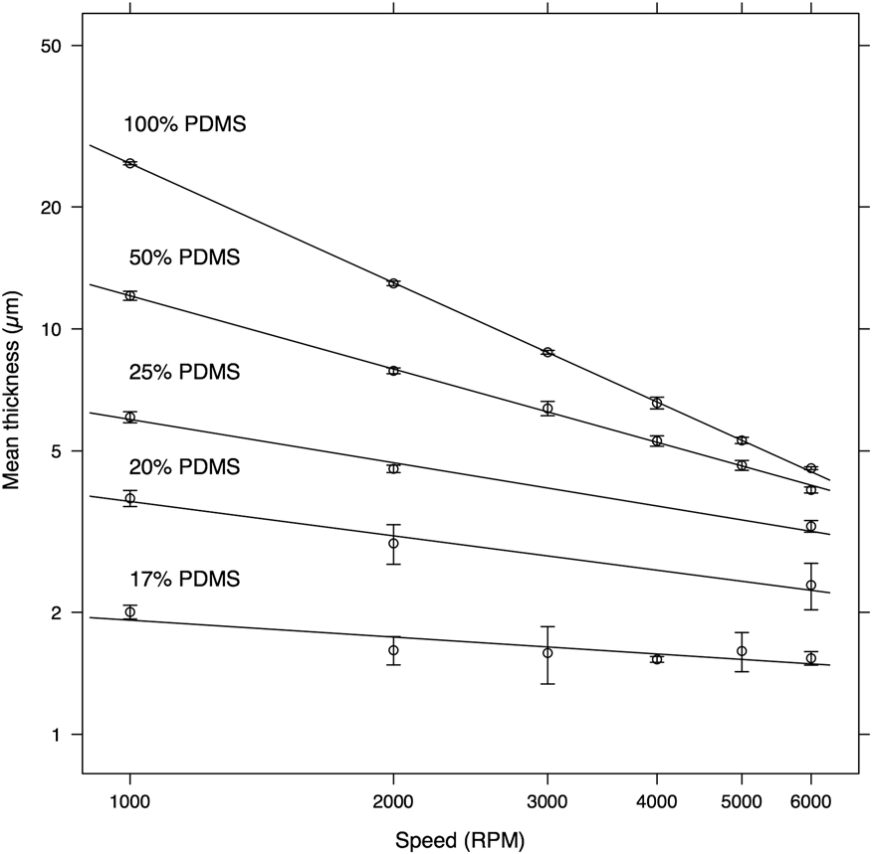
Thickness of the PDMS film under various concentrations (by weight) of PDMS in tert-butyl alcohol (TBA) as a function of spin speed. Each data point is the average of the mean thickness of three slides. Each slide was spun for 5 min. The error bar is the 95% confidence interval. The line is the least-squares fit of the data. The density of TBA is 0.775 g/mL at 25°C.

Figure 2 shows the PDMS film thickness for different concentrations of PDMS in TBA as a function of spin speed. Each line in Figure 2 is a fit of the data to the generally accepted relationship between angular velocity, *ω*, and thickness, *h* [8], [9]:
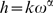
(1)where α and *k* are the experimentally derived constants shown in Table 2.

**Table 2:** Constants in the mathematical relationship between spin speed and PDMS film thickness.

Concentration of PDMS in TBA by weight	α	*k*
100%	−0.98	22,000
50%	−0.60	760
33%	−0.35	69
25%	−0.281	26.3
17%	−0.138	4.97

The parameters α and *k* are derived from the least-squares fit of the data summarized in Figure 2 using Equation 1 where angular velocity is in RPM and thickness is in µm.

**Figure 3:**
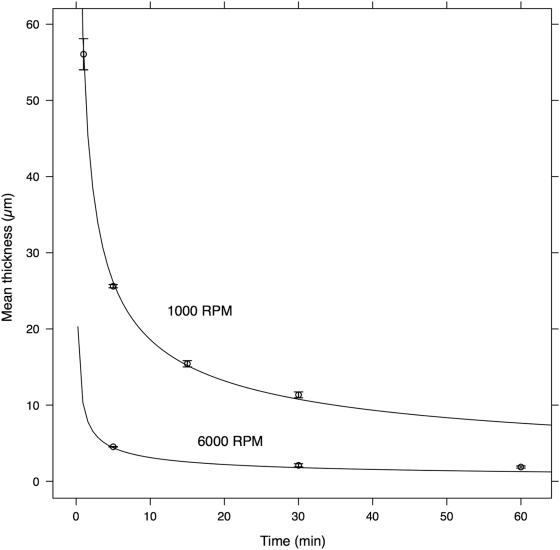
Thickness of the 100% PDMS film under two different spin speeds as a function of spin time. Each data point is the average of the mean thickness of three slides. The error bar is the 95% confidence interval. Each curve is a plot of Equation (2) for the given speed.

The thickness of the PDMS film for longer spin times is shown in Figure 3. The PDMS was not diluted in TBA for these measurements. Each line in Figure 3 is a plot of the following relationship, theoretically derived by Emslie et al.[10], between spin time, *t*, angular velocity, ω, and thickness, *h*:
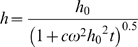
(2)where *h*_0_ and *c* have been experimentally derived here to be *h*_0_ = 180 µm and *c* = 2.86×10^−10^ RPM^−2^ µm^−2^ s^−1^.

The PDMS film spun onto on a PDMS substrate was found to be thicker than the PDMS film spun onto a glass substrate. Table 3 shows the comparison for three different concentrations of PDMS in TBA.

**Table 3:** Thickness of PDMS films spun on glass substrates vs. PDMS substrates.

Percent PDMS	Mean thickness on glass (µm)	Mean thickness on PDMS (µm)	Increase in thickness from glass to PDMS (µm)	Percent increase in mean thickness from glass to PDMS
100%	12.8–13.1	14.3–14.6	1.3–1.6	11.3%
50%	7.8–8.0	8.7–9.2	0.9–1.3	14%
17%	1.48–1.74	1.68–1.92	0.07–0.30	11.6%

All spins were done for 5 min at 2000 RPM at the given concentration. The values are given as 95% confidence intervals for a sample of three slides.

**Figure 4:**
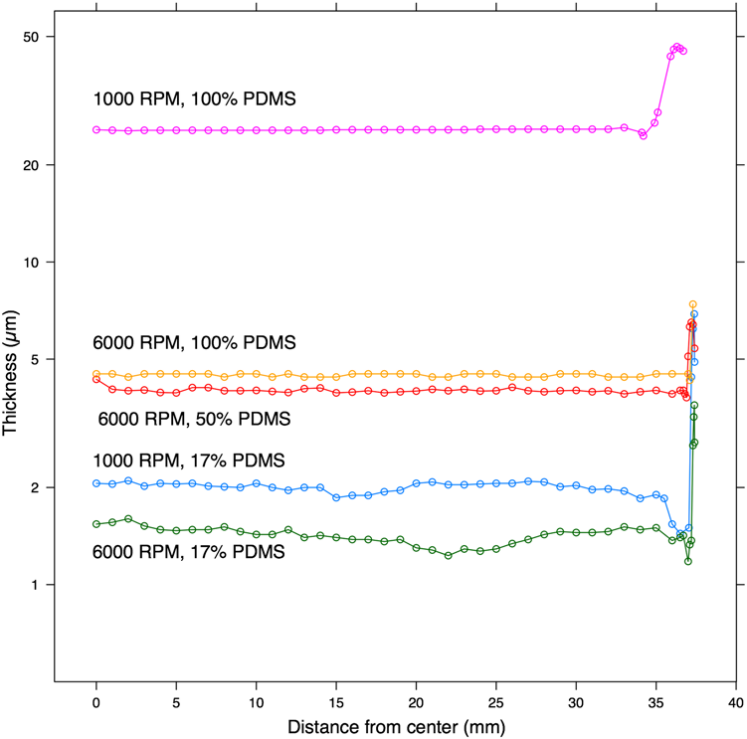
Profiles of five PDMS membranes spun for 5 min on glass. Each profile is a single sample, ending in the edge bead. The profiles in Figure 4 were measured from the center point of the slide to the edge along the long axis as indicated by the “Profile line” in Figure 5.

Figure 4 shows the profile of the membrane for different concentrations of PDMS at 1000 RPM and 6000 RPM. A strong edge bead was present in all cases.

Biocompatibility of the film was verified when single yeast cells were grown in yeast growth media on 10 different TBA-diluted films for 8 hours under a microscope. Each time, a normal ∼90 minute budding cycle was achieved within 3 hours of placing the cells on the film.

## Discussion

We have presented two alternatives to hexane dilution of PDMS: long spin times and dilution in tert-butyl alcohol (TBA). We have found that TBA is an excellent solvent for PDMS because it does not swell the underlying PDMS layer, and it dissolves uncured PDMS when it is mixed at 45°C. We have used dilution in TBA extensively in our lab to construct multilayer PDMS devices. For example, TBA-dissolved PDMS has served as a protective layer for single-cell magnetic trapping elements [7] and as a variable-resistance layer in fabricating thin-film thermocouples [11].

## Materials and Methods

To measure swelling ratios, PDMS part A and part B (Sylgard 184, Dow Corning Corp., Midland, MI) were mixed in a 10∶1 (weight∶weight) ratio and cured for three hours at 55°C in a 85 mm×125 mm×7 mm rectangular mold. 12 identical pieces were cut from the mold. The thickness (∼7 mm) of each piece was measured in three locations with a micrometer. Four pieces were placed into each solvent: deionized water at room temperature, TBA (Sigma-Aldrich, St. Louis, MO) at 45°C, and hexane (Sigma-Aldrich) at room temperature, and allowed to soak for two hours. The pieces were removed from the solvent, dried, and immediately measured with a micrometer as before.

All spin coat tests were performed on one of two substrates: a 2″×3″ glass slide, or a 2″×3″ glass slide coated with PDMS. The bare glass was rinsed in ethanol and deionized (DI) water. The PDMS membrane was rinsed in DI water only. The substrate was blown dry using nitrogen, and then dried on a 60 C hotplate for 30 minutes. It was then allowed to cool to room temperature and placed on the spinner (WS-400B-6NPP-Lite, Laurell Technologies Corp., North Wales, PA). PDMS part A and part B (Sylgard 184, Dow Corning Corp., Midland, MI) were then mixed in a 10∶1 (weight∶weight) ratio in a 25 ml polystyrene beaker using a glass stirring rod for 2 minutes. The PDMS was placed in a vacuum desiccator until bubbles were no longer visible (10–13 minutes). The PDMS was then mixed with 45°C TBA in the appropriate concentration until fully dissolved (30–45 s). The TBA needs to be warmed to 45°C for better mixing with the PDMS; TBA is a solid at room temperature. The mixture was then poured onto the glass slide. The total time between combining PDMS parts and starting the spin was exactly 15 min for each sample. The spinner was then started and then run for the desired time. Finally, the PDMS was placed on a 60 C hotplate until fully cured (1–2 hours).

**Figure 5:**
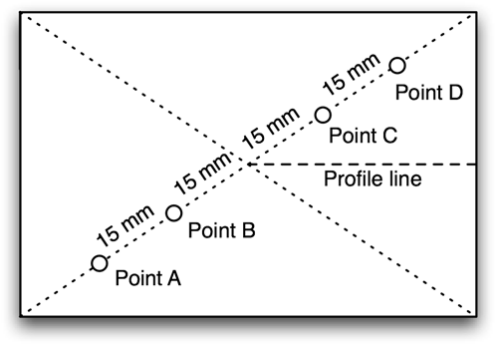
Diagram of points on the slide were thickness measurements were taken. The thickness of the film on each slide was calculated as the mean of the thicknesses of points A–D. The profile was generated by measuring the thickness at points spaced 1 mm apart (or less near the edge) along the profile line.

To measure the thickness of the membrane, the area of the PDMS to be profiled was scraped away with a clean razor blade. Both the PDMS and the glass in the area of the removed PDMS were then briefly electrolessly plated in silver per manufacturer's instructions (LI Silver, Nanoprobes Inc., Yaphank, NY) [12] so that the surfaces would be visible to the optical profilometer. The height of the silver plating was measured with AFM (Dimension 3100 Scanning Probe Microscope, Veeco, Woodbury, NY) to be less than 50 nm on both glass and PDMS (data not shown). The plated area was then profiled using an optical profilometer (Wyco NT3300, Veeco Instruments Inc., Woodbury, NY) to find the difference in height between the PDMS film and the substrate. Measurements less than 4.5 µm were made to the nearest 0.01 µm and measurements greater than 4.5 µm were made to the nearest 0.1 µm. Four locations were measured along the diagonal of each slide as shown in Figure 5. These four locations were averaged to generate a mean thickness per slide. The thickness of the membrane spun onto a PDMS substrate was measured by first measuring the thickness of the membrane on glass only and then measuring the mirror of points A–D (Figure 5) for the total membrane thickness. The mean thickness of PDMS-only membrane was calculated by subtracting the mean thickness of glass-only membrane from the mean thickness of combined membrane. Each 95% confidence interval in Figure 2 and Table 3 was calculated using a one or two sample (as appropriate) t-test of 3 samples, where each sample was the mean thickness per slide. The profiles in Figure 4 were measured from the center point of the slide to the edge along the long axis as shown in Figure 5.

Data analysis was performed using the R programming language. Estimates of the parameters in Equations (1) and (2) were found using the non-linear least squares (nls) function.
